# The Added Benefit of Opicapone When Used Early in Parkinson's Disease Patients With Levodopa-Induced Motor Fluctuations: A *Post-hoc* Analysis of BIPARK-I and -II

**DOI:** 10.3389/fneur.2021.754016

**Published:** 2021-11-05

**Authors:** José-Francisco Rocha, Georg Ebersbach, Andrew Lees, Eduardo Tolosa, Joaquim J. Ferreira, Werner Poewe, Olivier Rascol, Fabrizio Stocchi, Angelo Antonini, Diogo Magalhães, Helena Gama, Patrício Soares-da-Silva

**Affiliations:** ^1^BIAL – Portela & C^a^, S.A., Coronado, Portugal; ^2^Movement Disorders Clinic, Beelitz-Heilstätten, Germany; ^3^National Hospital for Neurology and Neurosurgery, London, United Kingdom; ^4^Parkinson Disease and Movement Disorder Unit, Neurology Service, Hospital Clínic de Barcelona, Institut d'Investigacions Biomèdiques August Pi i Sunyer (IDIBAPS), University of Barcelona (UB), Centro de Investigación Biomédica en Red sobre Enfermedades Neurodegenerativas (CIBERNED), Barcelona, Spain; ^5^Laboratory of Clinical Pharmacology and Therapeutics, Faculty of Medicine, University of Lisbon, Lisbon, Portugal; ^6^Department of Neurology, Medical University of Innsbruck, Innsbruck, Austria; ^7^Toulouse Parkinson's Expert Center, Departments of Neurosciences and Clinical Pharmacology, Centre d'Investigation Clinique de Toulouse CIC 1436, NS-Park/FCRIN Network, and NeuroToul COEN Center, University Hospital of Toulouse, INSERM, University of Toulouse 3, Toulouse, France; ^8^Department of Neurology, IRCCS San Raffaele Pisana, Rome, Italy; ^9^Parkinson and Movement Disorders Unit, Center for Neurodegenerative Disease (CESNE), Department of Neurosciences, University of Padova, Padova, Italy

**Keywords:** catechol-O-methyltransferase inhibitor, levodopa, motor fluctuations, opicapone, Parkinson's disease, wearing-off

## Abstract

**Introduction:** Opicapone (OPC) was efficacious in reducing OFF-time in two pivotal trials in patients with Parkinson's disease (PD) and end-of-dose motor fluctuations (BIPARK-I and -II). *Post-hoc* analyses of these trials evaluated the efficacy of OPC following pre-defined segmentation of the wide spectrum of motor fluctuations in PD.

**Methods:** Data from matching treatment arms in BIPARK-I and -II were combined for the placebo (PLC) and OPC 50-mg groups, and exploratory *post-hoc* analyses were performed to investigate the efficacy of OPC 50 mg vs. PLC in subgroups of patients who were in “earlier” vs. “later” stages of both their disease course (e.g., duration of PD <6 years vs. ≥6 years) and levodopa treatment pathway (e.g., number of daily levodopa intakes <4 vs. ≥4). Efficacy variables included changes from baseline in absolute OFF-time and total ON-time.

**Results:** The Full Analysis Set included 517 patients (PLC, *n* = 255; OPC 50 mg, *n* = 262). OPC 50 mg was significantly more effective than PLC in reducing OFF-time and increasing ON-time in the majority of subgroup analyses (*p* < 0.05). Moreover, patients in “earlier” stages of both their disease course and levodopa treatment pathway experienced numerically greater efficacy when using OPC 50 mg, in comparison with those in “later” stages.

**Conclusion:** OPC 50 mg was efficacious over the whole trajectory of motor fluctuation evolution in PD patients. There was also a signal for enhanced efficacy in patients who were earlier vs. later in their disease course and levodopa treatment pathway.

## Introduction

More than 50 years since its introduction, levodopa (L-DOPA) remains the most efficacious treatment for Parkinson's disease (PD) (1). The long-term success of L-DOPA is compromised by the development of motor complications, but recent studies have shown that delaying the initiation of L-DOPA results in a reduced quality of motor control that is not offset by longer-term benefits (2–5). Indeed, longer disease duration at the start of L-DOPA therapy is an independent and important risk factor for the development of motor fluctuations and dyskinesias, as is the dose (but not the duration) of L-DOPA used (6, 7).

It has been proposed that the emergence of response fluctuations and drug-induced dyskinesias in the course of sustained treatment with L-DOPA results from discontinuous drug delivery and pulsatile stimulation of striatal dopamine receptors, which result in downstream changes in the basal ganglia (8, 9). Furthermore, response fluctuations are attributed to increasing loss of buffering capacity in progressively diminishing neurons (9). Hypothetically, improving bioavailability and steadiness of exogenous L-DOPA may result in a more extended ON-time period and less troublesome dyskinesia in patients in early stages of PD when the pulsatile stimulation of the system is not yet severe, and the priming effect is less profound compared to patients with more advanced disease. Once established, such motor complications can be difficult to treat, but a variety of pharmacological and non-pharmacological interventions have shown efficacy in clinical trials (10, 11). A common initial approach to wearing-off effects is to modify the administration of L-DOPA, often by using smaller, more frequent doses of L-DOPA, increasing the total dose of L-DOPA, or switching to controlled-release or modified-release L-DOPA preparations. In most patients, these strategies are at best successful for a year or two (12, 13). Prolongation of the clinical effect of L-DOPA by co-administering with a long-acting dopamine agonist (DA) (14) or catechol-O-methyltransferase (COMT) inhibitor (15), or by preventing dopamine degradation in the brain with a selective monoamine oxidase inhibitor (MAO-BI) (16), are other effective strategies.

COMT inhibitors extend the half-life and bioavailability of L-DOPA and may lead to a more continuous delivery of L-DOPA to the brain (15). Opicapone (OPC) is a third-generation, once-daily COMT inhibitor developed to fulfill the need for a more potent, longer-acting COMT inhibitor, with a well-established safety profile (17–20). OPC has been shown to be generally well-tolerated and efficacious in reducing OFF-time in two pivotal trials in patients with PD and end-of-dose motor fluctuations (BIPARK-I and -II) (21, 22). On the basis of these trials, OPC was first approved in the European Union as adjunctive therapy to preparations of L-DOPA/dopa decarboxylase inhibitors in adult patients with PD and end-of-dose motor fluctuations who cannot be stabilized on those combinations (23). Presently, it is also approved and marketed in the USA, Japan, South Korea, Australia, and other countries.

We have now conducted exploratory *post-hoc* analyses of data from the BIPARK-I and -II trials (21, 22) to evaluate the efficacy of OPC following a pre-defined segmentation of the wide spectrum of motor fluctuations in PD, based on baseline disease- and therapy-related characteristics.

## Materials and Methods

### Study Design

**Figure 1:**
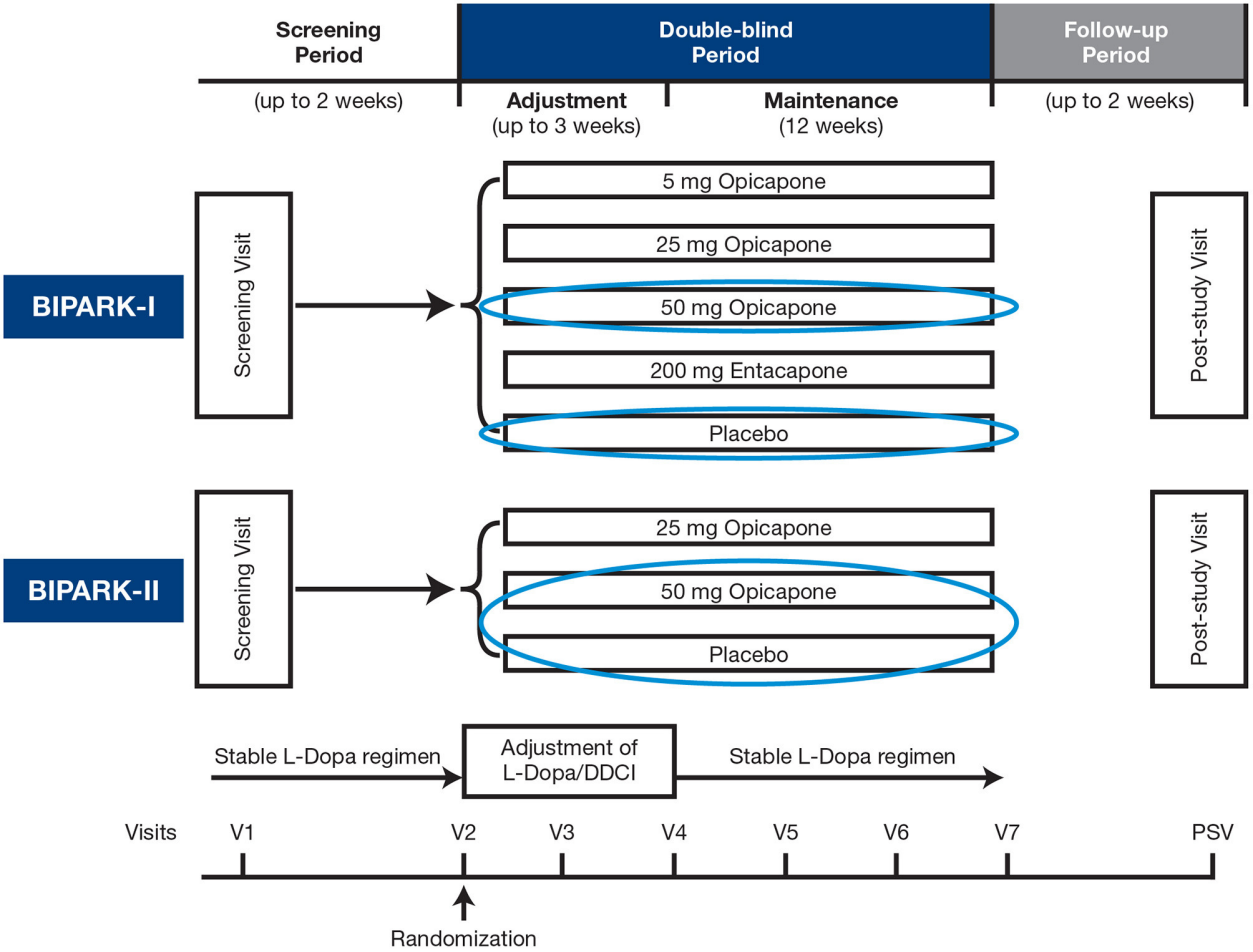
Study design. DDCI, dopa decarboxylase inhibitor; L-Dopa, levodopa; PSV, post-study visit; V, visit.

BIPARK-I and -II were Phase III, multicenter, randomized, double-blind, placebo (PLC)-controlled trials of OPC as an adjunct to L-DOPA in patients with PD with end-of-dose motor fluctuations, details of which have been published previously (21, 22). The trials had similar designs (Figure 1), eligibility criteria, and methods. In BIPARK-I, patients were randomized to treatment with OPC (5, 25, or 50 mg once daily), PLC, or entacapone (200 mg with every L-DOPA intake) for 14–15 weeks (21). In BIPARK-II, patients were randomized to treatment with OPC (25 or 50 mg once daily) or PLC for 14–15 weeks (22). In both trials, the primary efficacy endpoint was change from baseline to endpoint in absolute OFF-time vs. PLC, based on patient diaries (21, 22).

In the current study, data from matching treatment arms in BIPARK-I and -II were combined for the PLC and OPC 50-mg groups and exploratory *post-hoc* analyses were performed to investigate the efficacy and safety/tolerability of OPC 50 mg vs. PLC in patients who were divided on the basis of baseline disease- and therapy-related characteristics into representative subgroups of patients who were in “earlier” or “later” stages of both their disease course and L-DOPA treatment pathway, within the motor fluctuations spectrum of PD.

### Study Population

In BIPARK-I and -II, eligible patients were male or female, aged 30–83 years, with a ≥3-year diagnosis of idiopathic PD, Hoehn and Yahr (H&Y) 1–3 at ON-state, who were receiving L-DOPA treatment for ≥1 year and experiencing end-of-dose motor fluctuations. Details of the full inclusion/exclusion criteria from the trials have been published previously (21, 22). These *post-hoc* analyses included all patients treated with OPC 50 mg and PLC in BIPARK-I and -II.

### Study Assessments

Baseline characteristics, efficacy, and safety/tolerability were assessed for each patient pairwise baseline subgroup, defined on the basis of a putative segmentation of the motor fluctuations spectrum, for both disease- and therapy-related characteristics. Disease-related characteristics comprised duration of PD (<6 years vs. ≥6 years; <7 years vs. ≥7 years; <8 years vs. ≥8 years; <9 years vs. ≥9 years), H&Y staging (<2.5 vs. ≥2.5), and timing of onset of motor fluctuations ( ≤ 1 year [termed “recent motor fluctuators”] vs. >1 year; ≤ 2 years [termed “early motor fluctuators”] vs. >2 years). Treatment-related characteristics consisted of number of L-DOPA intakes (<4 vs. ≥4; <5 vs. ≥5; <6 vs. ≥6), L-DOPA daily amount (<500 vs. ≥500 mg; <600 vs. ≥600 mg; <700 vs. ≥700 mg; <800 vs. ≥800 mg), use of L-DOPA only (i.e., without a DA or MAO-BI) (Yes vs. No), use of L-DOPA plus a DA (Yes vs. No), and use of L-DOPA plus a MAO-BI (Yes vs. No). Baseline characteristics were summarized for the above subgroups and included age, sex, absolute OFF-time, duration of PD, time since onset of motor fluctuations, H&Y staging at ON, L-DOPA daily dose, and duration of L-DOPA therapy.

Efficacy variables consisted of absolute OFF-time, total ON-time, and ON-time with troublesome dyskinesia, evaluated in patients treated with OPC 50 mg or PLC. Safety/tolerability is not addressed here as it is planned to publish this separately.

### Statistical Analyses

Patient disposition and demographic/baseline characteristics were assessed for the Safety Set, which included all patients who received at least one dose of study drug. Efficacy assessments were conducted for the Full Analysis Set (FAS), which included all randomly assigned patients who took at least one dose of study drug and had at least one post-baseline efficacy assessment.

Subgroup analyses were performed *via* an analysis of covariance (ANCOVA) that modeled the change of each efficacy variable from baseline to endpoint as a linear fixed-effect model of study and geographical area as factors and baseline respective pairwise variables as covariate in the FAS. Each pairwise comparison was analyzed separately, so multiple comparison correction was not required. Ninety-five percent confidence intervals and matching *p*-values were derived for the least square (LS) mean estimates and their differences. The last observation carried forward (LOCF) was applied to handle missing diary data. Forest plots are presented to visually assess differentiation for each pairwise subgroup.

## Results

### Study Population

**Figure 2:**
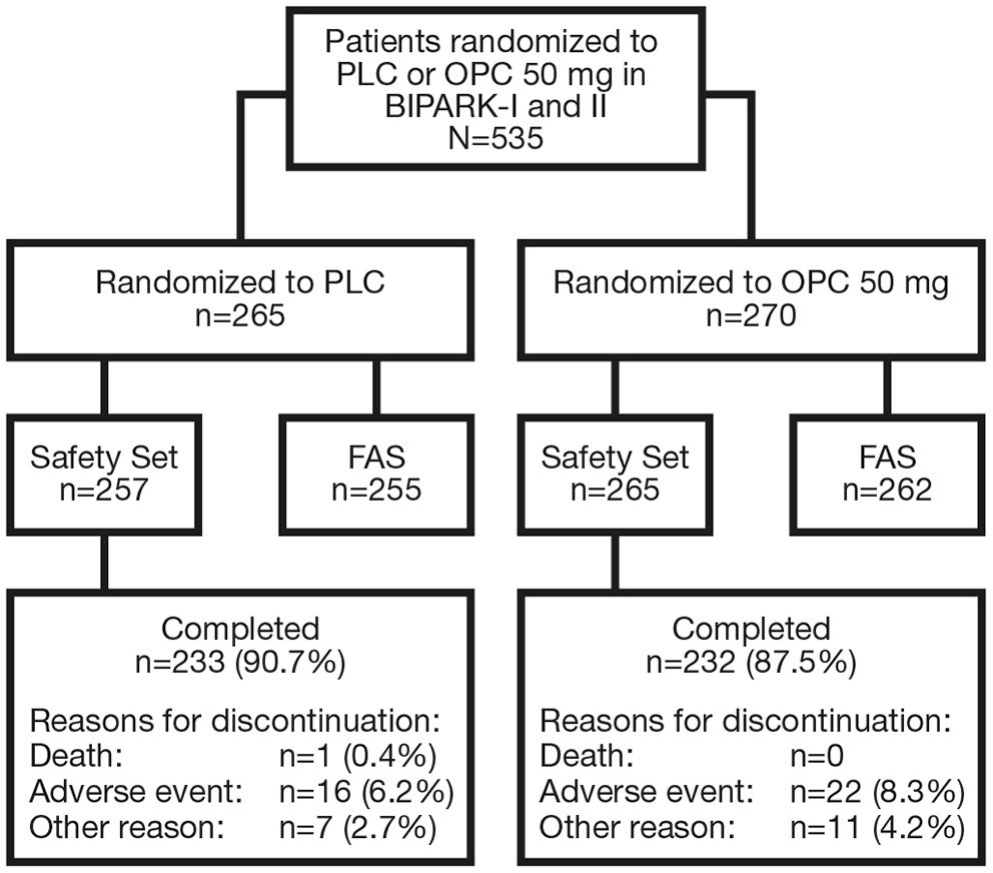
Flowchart of patient disposition. FAS, full analysis set; OPC, opicapone; PLC, placebo.

In total, 535 patients were randomized to receive PLC or OPC 50 mg in BIPARK-I and -II (Figure 2). The Safety Set included 522 patients (PLC, *n* = 257; OPC 50 mg, *n* = 265) and the FAS included 517 patients (PLC, *n* = 255; OPC 50 mg, *n* = 262). In the overall OPC 50 mg Safety Set, 60.4% of patients were male, mean (standard deviation [SD]) age was 64.5 (8.8) years, mean (SD) duration of PD was 7.6 (4.3) years, mean (SD) time since onset of motor fluctuations was 2.7 (2.9) years, mean (SD) H&Y staging at ON was 2.4 (0.5), mean (SD) absolute OFF-time at baseline was 6.2 (2.0) h, mean (SD) L-DOPA dose at baseline was 698.4 (322.1) mg/day, and mean (SD) duration of L-DOPA therapy was 6.3 (4.4) years. Baseline characteristics of the overall PLC Safety Set were similar to the OPC 50 mg Safety Set (24). Baseline characteristics by OPC 50 mg and PLC subgroups are summarized in Supplementary Tables 1, 2, respectively.

### Efficacy

**Figure 3:**
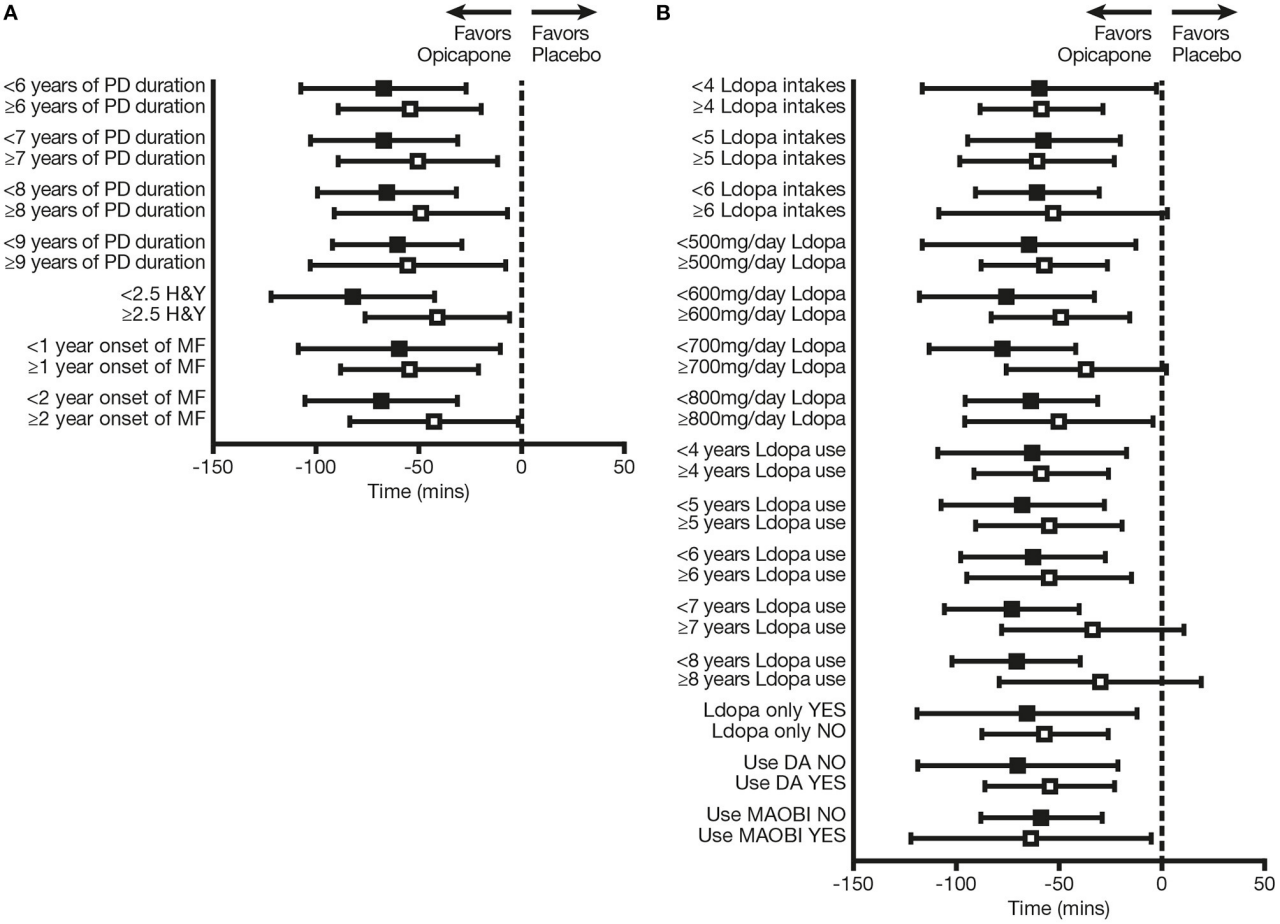
Change from baseline in absolute OFF-time in subgroups of patients defined on the basis of **(A)** baseline disease-related characteristics and **(B)** baseline therapy-related characteristics. Black squares indicate subgroups of patients who were “earlier” in their disease course and L-DOPA treatment pathway; open squares indicate the corresponding comparator subgroups of patients who were “later” in their disease course and L-DOPA treatment pathway. DA, dopamine agonist; H&Y, Hoehn and Yahr; L-DOPA, levodopa; MAO-BI, monoamine oxidase-B inhibitor; MF, motor fluctuations; PD, Parkinson's disease.

OPC 50 mg was significantly more effective than PLC in reducing OFF-time from baseline in the majority of subgroup analyses (*p* < 0.05), the exceptions being patients who received ≥6 L-DOPA intakes (*p* = 0.0623), patients with L-DOPA treatment duration ≥7 years (*p* = 0.1352), patients with L-DOPA treatment duration ≥8 years (*p* = 0.2309), and patients treated with ≥700 mg/day L-DOPA (*p* = 0.0640) (Table 1; Figure 3). Moreover, patients who were in “earlier” stages of both their disease course and L-DOPA treatment pathway experienced numerically greater efficacy when using OPC 50 mg, in comparison with those in “later” phases. OPC 50 mg demonstrated greater efficacy vs. PLC in each pairwise subgroup, with the following two exceptions: patients who received <5 L-DOPA intakes vs. ≥5 L-DOPA intakes (−57.5 vs. −60.8 min) and patients who received L-DOPA without an MAO-BI vs. those who received L-DOPA plus an MAO-BI (−58.6 vs. −63.7 min) (Table 1; Figure 3). Nevertheless, the OPC 50 mg magnitude of effect for these two exceptions was greater in each pairwise subgroup of patients who were in “earlier” phases of their motor fluctuation trajectory.

**Table 1:** Change from baseline in absolute OFF-time by subgroup (FAS).

**Subgroup**		**OPC**		**PLC**		**OPC vs. PLC aannggeell Δ (SE) change from baseline (min)**	***p*-value**
		***N***	**LS mean (SE) change from baselineaannggeell (min)**	***N***	**LS mean (SE)change from baseline aannggeell(min)**		
**Disease-related subgroups**							
Duration of PD (years)	<6	117	−109.1 (14.4)	102	−41.7 (15.2)	**−67.5 (20.5)**	0.0011
	≥6	145	−122.7 (13.1)	153	−68.2 (12.7)	−54.5 (17.8)	0.0022
	<7	144	−116.1 (13.0)	133	−49.0 (13.4)	**−67.1 (18.2)**	0.0003
	≥7	118	−117.2 (14.6)	122	−66.8 (14.1)	−50.5 (19.8)	0.0110
	<8	159	−117.3 (12.4)	154	−51.6 (12.6)	**−65.7 (17.2)**	0.0001
	≥8	103	−115.5 (15.7)	101	−66.5 (15.5)	−49.0 (21.5)	0.0229
	<9	179	−114.4 (11.7)	179	−53.9 (11.7)	**−60.5 (16.1)**	0.0002
	≥9	83	−121.1 (17.2)	76	−65.6 (17.8)	−55.5 (24.3)	0.0226
H&Y staging	<2.5	113	−124.7 (14.6)	113	−42.6 (14.4)	**−82.1 (20.3)**	<0.0001
	≥2.5	149	−110.6 (12.9)	142	−69.5 (13.4)	−41.1 (17.8)	0.0214
Onset of MF (years)	≤ 1	85	−134.2 (17.2)	71	−74.4 (18.6)	**−59.7 (25.0)**	0.0173
	>1	161	−108.0 (12.6)	172	−53.3 (12.3)	−54.7 (17.0)	0.0014
	≤ 2	142	−127.1 (13.3)	125	−58.5 (14.1)	**−68.5 (18.9)**	0.0003
	>2	104	−103.7 (15.6)	118	−61.0 (14.7)	−42.7 (20.9)	0.0416
**Therapy-related subgroups**							
L-DOPA intakes (n)	<4	60	**–**124.5 (20.1)	51	−64.7 (21.5)	**−59.7 (29.0)**	0.0397
	≥4	202	−114.1 (11.1)	204	−55.5 (11.1)	−58.6 (15.2)	0.0001
	<5	130	−118.2 (13.7)	130	−60.7 (13.6)	−57.5 (18.9)	0.0024
	≥5	132	−114.7 (13.7)	125	−53.9 (14.0)	**−60.8 (19.1)**	0.0016
	<6	202	−120.6 (11.0)	195	−60.0 (11.3)	**−60.6 (15.3)**	<0.0001
	≥6	60	−101.6 (20.4)	60	−48.7 (20.6)	−52.9 (28.4)	0.0623
L-DOPA duration (years)	<4	96	−107.8 (15.9)	77	−44.6 (17.5)	**−63.2 (23.4)**	0.0070
	≥4	166	−121.7 (12.4)	178	−62.9 (11.8)	−58.8 (16.6)	0.0004
	<5	124	−107.9 (13.9)	104	−40.2 (15.1)	**−67.8 (20.2)**	0.0008
	≥5	138	−124.6 (13.5)	151	−69.5 (12.8)	−55.0 (18.1)	0.0024
	<6	149	−113.4 (12.7)	140	−50.8 (13.2)	**−62.7 (17.9)**	0.0005
	≥6	113	−120.5 (14.9)	115	−65.5 (14.6)	−55.0 (20.4)	0.0072
	<7	171	−122.8 (11.9)	160	−49.7 (12.4)	**−73.0 (16.7)**	<0.0001
	≥7	91	−104.0 (16.6)	95	−70.3 (16.0)	−33.7 (22.5)	0.1352
	<8	187	−122.2 (11.4)	179	−51.4 (11.7)	**−70.8 (15.9)**	<0.0001
	≥8	75	−101.7 (18.3)	76	−71.8 (17.8)	−30.0 (25.0)	0.2309
L-DOPA daily amount (mg)	<500	65	−118.9 (19.2)	68	−54.3 (18.8)	**−64.6 (26.4)**	0.0146
	≥500	197	−115.6 (11.3)	187	−58.5 (11.5)	−57.2 (15.6)	0.0003
	<600	102	−114.2 (15.3)	97	−38.7 (15.8)	**−75.5 (21.6)**	0.0005
	≥600	160	−118.1 (12.5)	158	−68.9 (12.5)	−49.2 (17.2)	0.0042
	<700	143	−124.9 (13.1)	138	−47.3 (13.3)	**−77.6 (18.2)**	<0.0001
	≥700	119	−106.5 (14.3)	117	−69.6 (14.4)	−36.9 (19.9)	0.0640
	<800	175	−113.5 (11.9)	170	−50.0 (12.0)	**−63.6 (16.4)**	0.0001
	≥800	87	−122.7 (16.7)	85	−72.5 (16.8)	−50.2 (23.3)	0.0316
Use of L-DOPA only	Yes	67	−108.3 (18.9)	59	−42.7 (20.0)	**−65.6 (27.2)**	0.0163
	No	195	−119.4 (11.4)	196	−62.2 (11.3)	−57.2 (15.5)	0.0002
Use of L-DOPA plus DA	Yes	178	−114.9 (12.0)	185	−60.3 (11.6)	−54.6 (16.0)	0.0007
	No	84	−119.8 (16.9)	70	−49.7 (18.4)	**−70.1 (24.8)**	0.0047
Use of L-DOPA plus MAO-BI	Yes	56	−105.4 (20.6)	49	−41.7 (22.4)	**−63.7 (29.8)**	0.0326
	No	206	−119.6 (11.1)	206	−61.1 (10.9)	−58.6 (15.0)	0.0001

*Rows shaded in gray indicate variables generally associated with earlier disease course (shorter PD duration, lower H&Y staging, and shorter onset of MF; lower number of L-DOPA intakes, shorter duration of L-DOPA use, lower daily L-DOPA dose amount, and less use of adjunctive therapies), in comparison with matched unshaded rows. Values shown in bold indicate variables for which the difference in change from baseline in OFF-time for OPC 50 mg vs. PLC (Δ) was greater than that of the matched comparative row*.

*DA, dopamine agonist; FAS, Full Analysis Set; H&Y, Hoehn and Yahr; L-DOPA, levodopa; LS, least square; MAO-BI, monoamine oxidase B inhibitor; MF, motor fluctuations; OPC, opicapone; PD, Parkinson's disease; PLC, placebo; SE, standard error*.

OPC 50 mg was also significantly more effective than PLC in increasing total ON-time from baseline in the majority of subgroup analyses (*p* < 0.05), excluding the following: patients with duration of PD ≥8 years (*p* = 0.0541), patients with onset of motor fluctuations >2 years previously (*p* = 0.0527), patients who received ≥6 L-DOPA intakes (*p* = 0.0767), patients with L-DOPA treatment duration ≥7 years (*p* = 0.4855), and patients with L-DOPA treatment duration ≥8 years (*p* = 0.4902) (Supplementary Table 3). As for OFF-time reduction, patients who were “earlier” regarding both their disease course and L-DOPA treatment pathway experienced numerically greater efficacy when using OPC 50 mg, in comparison with those in “later” phases. OPC 50 mg demonstrated enhanced efficacy vs. PLC in each pairwise subgroup, except for patients who received L-DOPA without an MAO-BI vs. those who received L-DOPA plus an MAO-BI (59.8 vs. 77.7 min) (Supplementary Table 3). Nevertheless, the OPC 50 mg magnitude of effect even for this exception was greater in the pairwise subgroup of patients who were in “earlier” phases.

Increases from baseline in ON-time with troublesome dyskinesia were not significantly greater for OPC 50 mg in comparison with PLC in all subgroup analyses (*p* ≥ 0.05), with the following exceptions: patients who received ≥5 L-DOPA intakes (*p* = 0.0095), patients with L-DOPA treatment duration ≥4 years (*p* = 0.0295), and patients with L-DOPA treatment duration ≥6 years (*p* = 0.0148)—all in the pairwise subgroups of patients who were in “later” phases (Supplementary Table 4). Moreover, differences between OPC 50 mg vs. PLC in the increase from baseline in ON-time with troublesome dyskinesia were less in the majority of subgroups of patients who were “earlier” vs. “later” in both their disease course and L-DOPA treatment pathway, the exceptions being the following: patients with PD duration <9 vs ≥9 years (11.1 vs. 10.7 min), patients with L-DOPA treatment duration <8 vs. ≥8 years (12.4 vs. 8.8 min), patients whose daily L-DOPA amount was <700 vs. ≥700 mg (13.0 vs. 9.5 min), and patients who received L-DOPA without a DA vs. those who received L-DOPA plus a DA (12.0 vs. 11.1 min) (Supplementary Table 4). Nevertheless, none of the differences were more than 5 min between each pairwise subgroup.

## Discussion

These exploratory *post-hoc* analyses of BIPARK-I and -II demonstrated that OPC 50 mg is efficacious over the whole trajectory of motor fluctuation evolution in PD patients, with similar effect sizes in subjects with recent onset of wearing-off effects and those in more advanced stages. OPC 50 mg was significantly more effective than PLC in reducing OFF-time and increasing ON-time for nearly all the subgroups that were analyzed (*p* < 0.05). The patients with shorter disease duration and duration of motor fluctuations, and those who were relatively early in their L-DOPA treatment pathway, experienced greater efficacy when using OPC 50 mg than those with later PD stages. Even in the “early” subgroups for which statistical significance was not demonstrated, there was a trend toward superiority of OPC 50 mg over PLC (*p*-values between 0.05 and 0.1). Changes in ON-time with troublesome dyskinesia were small and did not differ significantly from PLC for nearly all subgroup analyses (*p* ≥ 0.05). Furthermore, differences between OPC 50 mg vs. PLC in the increase from baseline in ON-time with troublesome dyskinesia were less in most of the subgroups of patients who were “earlier” in both their disease course and L-DOPA treatment pathway. These findings not only indicate that OPC is efficacious across all stages of development of motor fluctuations in PD patients, but also that patients who are at an early stage of their disease course may especially benefit from its introduction.

L-DOPA is the most effective symptomatic treatment for PD from the early stages of the disease (2–4). The priority of treatment is therefore to obtain clinically meaningful benefit from each L-DOPA intake by facilitating its delivery to the brain. Optimization of the peripheral metabolism of L-DOPA through COMT inhibition is a rational first approach. When OPC is used, this also has the advantage of allowing a simplified drug regimen, since, unlike entacapone and tolcapone, OPC is administered once daily (20). In the prospective, multicenter, open-label OPTIPARK study, a total of 495 patients were treated with OPC 50 mg for 3 (Germany) or 6 (UK) months, in addition to their current L-DOPA and other anti-Parkinsonian treatments, and 393 (79.4%) patients completed 3 months of treatment (25). After 3 months, 71.3% of patients showed improvement on the Clinician's Global Impression of Change (primary endpoint) and 76.9% experienced improvement on the Patient Global Impressions of Change (25). These findings complement existing evidence from BIPARK-I and -II (21, 22), by demonstrating that the efficacy of OPC 50 mg observed in the clinical trials was also experienced by PD patients with motor fluctuations treated in everyday routine clinical practice.

The current study was exploratory in nature and involved a *post-hoc* analysis. The BIPARK trials were not powered for the subgroups included in the analysis and low patient numbers in some subgroups may have led to insufficient statistical power to detect differences. Moreover, there were differences in the magnitude of effect of both OPC and PLC between subgroups and it is therefore important to consider not only the overall treatment difference for OPC vs. PLC but also the magnitude of effect of both OPC and PLC when interpreting the findings for individual subgroups. Further analysis is planned to try to identify patient profile(s) that might particularly benefit (or not benefit) from OPC therapy.

In summary, this study supports the efficacy of OPC 50 mg, in comparison with PLC, across the entire trajectory of motor fluctuation development in PD, from very early fluctuation to those with more advanced stages. It also indicates that patients who were in “earlier” stages in relation to their disease duration and the time since first occurrence of motor fluctuations may have enhanced efficacy when using OPC; further work is required to establish this. The pathophysiological basis for this remains unclear but may relate to less advanced nigrostriatal denervation and less severe pulsatile stimulation of the system compared to later disease stages.

## Data Availability Statement

The original contributions presented in the study are included in the article/Supplementary Material, further inquiries can be directed to the corresponding author/s.

## Ethics Statement

The studies involving human participants were reviewed and approved by Institutional Review Boards at the participating sites (see Supplementary Material for full list). The patients/participants provided their written informed consent to participate in this study.

## Author Contributions

All authors listed have made a substantial, direct, and intellectual contribution to the work and approved it for publication.

## Funding

The study, data analysis, and manuscript preparation were funded by Bial – Portela & Cª, S.A.

## Conflict of Interest

J-FR is an employee of Bial – Portela & Cª, S.A. GE has received honoraria for advisory boards and consultancy from AbbVie Pharma, BIAL Pharma, Biogen GmbH, Desitin Pharma, STADA Pharma, and NeuroDerm Inc.; speaker's honoraria from AbbVie Pharma, BIAL Pharma, Britannia Pharma, Desitin Pharma, Licher GmbH, UCB Pharma, and Zambon Pharma; and royalties from Kohlhammer Verlag and Thieme Verlag. AL is funded by the Reta Lila Weston Institute of Neurological Studies, University College London, Institute of Neurology and reports consultancies from Britannia Pharmaceuticals and BIAL Portela. He also reports grants and/or research support from the Frances and Renee Hock Fund, and honoraria from Britannia Pharmaceuticals, BIAL, STADA, UCB, and Nordiclnfu Care. ET received honoraria for consultancy from TEVA, Bial, Prevail Therapeutics, Boehringer Ingelheim, Roche, and BIOGEN, and has received funding for research from the Spanish Network for Research on Neurodegenerative Disorders CIBERNED - Instituto Carlos III (ISCIII), and The Michael J. Fox Foundation for Parkinson's Research (MJFF). JF has provided consultancy for Ipsen, GlaxoSmithKline, Novartis, Teva, Lundbeck, Solvay, Abbott, BIAL, Merck-Serono, and Merz, and has received grants from GlaxoSmithKline, Grunenthal, Teva, and Fundação MSD. WP has received lecture fees and honoraria for consultancy in relation to clinical drug development programs from Alterity, AbbVie, Affiris, AstraZeneca, Axovant, BIAL, Biogen, Britannia, Lilly, Lundbeck, NeuroDerm, Neurocrine, Denali Pharmaceuticals, Orion Pharma, Roche, Stada, Sunovion, Takeda, UCB, and Zambon, as well as grant support from the MJFF and the EU FP7 & Horizon 2020 programs. OR has participated in advisory boards and/or provided consultancy for AbbVie, Adamas, Acorda, Addex, AlzProtect, ApoPharma, AstraZeneca, Axovant, Bial, Biogen, Britannia, Buckwang, CereSpir, Clevexel, Denali, INC Research, IPMDS, Lundbeck, Lupin, Merck, MundiPharma, NeurATRIS, NeuroDerm, Novartis, ONO Pharma, Osmotica, Parexel, Pfizer, Prexton Therapeutics, Quintiles, Roche, Sanofi, Servier, Sunovion, Theranexus, Takeda, Teva, UCB, Vectura, Watermark Research, XenoPort, XO, and Zambon; received grants from Agence Nationale de la Recherche (ANR), CHU de Toulouse, France-Parkinson's, INSERM-DHOS Recherche Clinique Translationnelle, MJFox Foundation, Programme Hospitalier de Recherche Clinique, European Commission (FP7, H2020), and Cure Parkinson's UK; and received a grant to participate in a symposium and contribute to the review of an article by the International Parkinson's and Movement Disorder Society. FS has received compensation for consultancy and speaker-related activities from Lundbeck, UCB, Chiesi, Zambon, Britannia, Cynapsus, Sunovion, Kyowa, Abbvie, Neuroderm, Biogen, and Bial. AA has received compensation for consultancy and speaker-related activities from UCB, Boehringer Ingelheim, Britannia, AbbVie, Zambon, Bial, NeuroDerm, Theravance Biopharma, and Roche; he receives research support from Chiesi Pharmaceuticals, Lundbeck, Horizon 2020 - Grant 825785, Horizon 2020 Grant 101016902, Ministry of Education University and Research (MIUR) Grant ARS01_01081, and Cariparo Foundation. He serves as consultant for Boehringer Ingelheim for legal cases on pathological gambling; owns Patent WO2015110261-A1; and owns shares in PD Neurotechnology Limited. DM, HG, and PS are the employee of Bial – Portela & Cª, S.A. The authors declare that this study received funding from BIAL – Portela & Cª, S.A. The funder was not involved in the study design, collection, analysis, interpretation of data, the writing of this article or the decision to submit it for publication.

## Publisher's Note

All claims expressed in this article are solely those of the authors and do not necessarily represent those of their affiliated organizations, or those of the publisher, the editors and the reviewers. Any product that may be evaluated in this article, or claim that may be made by its manufacturer, is not guaranteed or endorsed by the publisher.
