# Supplementary material for: The Added Benefit of Opicapone When Used Early in Parkinson's Disease Patients With Levodopa-Induced Motor Fluctuations: A Post-hoc Analysis of BIPARK-I and -II
Source: Front Neurol. 2021 Nov 5;12:754016. doi: 10.3389/fneur.2021.754016 (PMC8603564; doi:10.3389/fneur.2021.754016)
Supplement: Supplementary file 1 [file Data_Sheet_1.docx]

**The added benefit of opicapone when used early in Parkinson’s disease patients with levodopa-induced motor fluctuations: a *post-hoc* analysis of BIPARK-I and II**

**José‐Francisco Rocha,^1†^* Georg Ebersbach,^2†^ Andrew Lees,^3†^ Eduardo Tolosa,^4†^ Joaquim J. Ferreira,^5†^ Werner Poewe,^6†^ Olivier Rascol,^7†^ Fabrizio Stocchi,^8†^ Angelo Antonini,^9†^ Diogo Magalhães,^1†^ Helena Gama,^1†^ Patrício Soares-da-Silva^1†^**

^1^BIAL – Portela & Ca S.A., Coronado, Portugal; ^2^Movement Disorders Clinic, Beelitz-Heilstätten, Germany; ^3^National Hospital for Neurology and Neurosurgery, London, UK; ^4^Parkinson Disease and Movement Disorder Unit, Neurology Service, Hospital Clínic de Barcelona, Institut d'Investigacions Biomèdiques August Pi i Sunyer (IDIBAPS), University of Barcelona (UB), Centro de Investigación Biomédica en Red sobre Enfermedades Neurodegenerativas (CIBERNED) Barcelona, Spain; ^5^Laboratory of Clinical Pharmacology and Therapeutics, Faculty of Medicine, University of Lisbon, Lisbon, Portugal; ^6^Department of Neurology, Medical University of Innsbruck, Innsbruck, Austria; ^7^Toulouse Parkinson Expert Center, Departments of Neurosciences and Clinical Pharmacology, Centre d’Investigation Clinique de Toulouse CIC1436, NS-Park/FCRIN Network, and NeuroToul COEN Center, University Hospital of Toulouse, INSERM, University of Toulouse 3, Toulouse, France; ^8^Department of Neurology, IRCCS San Raffaele Pisana, Rome, Italy; ^9^Parkinson and Movement Disorders Unit, Center for Neurodegenerative disease (CESNE), Department of Neurosciences, University of Padova, Padova, Italy

^†^These authors have contributed equally to this work

***Corresponding author:**

José‐Francisco Rocha

BIAL – Portela & Ca S.A.,

Coronado,

Portugal

Tel: +351 22 986 6100

Email: [francisco.rocha@bial.com](mailto:francisco.rocha@bial.com)

**Supplementary Material**

**Supplementary Table 1. Baseline characteristics of OPC 50 mg patient subgroups (Safety Set)**

| **Subgroup** | | **N** | **Baseline** | | | | | | | |
| --- | --- | --- | --- | --- | --- | --- | --- | --- | --- | --- |
|  |  |  | **Age (mean [SD] years)** | **Duration of PD (mean [SD] years)** | **Absolute OFF-time (mean [SD] h)** | **Time since onset of motor fluctuations (mean [SD] years)** | **H&Y staging at ON (mean [SD])** | **Male gender (n [%])** | **L-DOPA dose (mean [SD] mg)** | **Duration of L-DOPA therapy (mean [SD] years)** |
| **Disease-related subgroups** | | | | | | | | | | |
| *Duration of PD (years)* | *<6* | 119 | 63.8 (9.5) | 4.3 (0.9) | 6.3 (1.8) | 1.4 (1.1) | 2.4 (0.6) | 81 (68.1) | 577.8 (301.8) | 3.5 (1.4) |
|  | *≥6* | 146 | 65.1 (8.3) | 10.4 (4.0) | 6.2 (2.2) | 3.9 (3.4) | 2.4 (0.5) | 79 (54.1) | 796.6 (305.2) | 8.6 (4.6) |
|  | *<7* | 146 | 63.9 (9.4) | 4.7 (1.2) | 6.2 (1.9) | 1.5 (1.1) | 2.4 (0.6) | 97 (66.4) | 615.1 (311.5) | 3.7 (1.6) |
|  | *≥7* | 119 | 65.3 (8.0) | 11.3 (3.9) | 6.3 (2.2) | 4.3 (3.6) | 2.5 (0.5) | 63 (52.9) | 800.6 (306.2) | 9.5 (4.6) |
|  | *<8* | 162 | 64.0 (9.2) | 5.0 (1.4) | 6.3 (2.0) | 1.6 (1.3) | 2.4 (0.6) | 105 (64.8) | 625.2 (306.4) | 3.9 (1.8) |
|  | *≥8* | 103 | 65.4 (8.1) | 11.9 (3.9) | 6.2 (2.1) | 4.5 (3.7) | 2.4 (0.5) | 55 (53.4) | 813.5 (313.9) | 10.1 (4.5) |
|  | *<9* | 182 | 64.2 (9.2) | 5.4 (1.7) | 6.3 (2.0) | 1.8 (1.5) | 2.4 (0.6) | 116 (63.7) | 652.5 (326.7) | 4.3 (2.1) |
|  | *≥9* | 83 | 65.1 (8.0) | 12.7 (3.9) | 6.0 (2.1) | 4.9 (3.9) | 2.4 (0.5) | 44 (53.0) | 799.1 (289.2) | 10.8 (4.8) |
| *H&Y staging* | *<2.5* | 113 | 63.0 (9.2) | 7.3 (4.3) | 6.1 (2.0) | 2.9 (3.1) | 1.9 (0.3) | 74 (65.5) | 678.3 (325.0) | 6.1 (4.3) |
|  | *≥2.5* | 152 | 65.6 (8.4) | 7.9 (4.3) | 6.4 (2.1) | 2.6 (2.7) | 2.8 (0.3) | 86 (56.6) | 713.3 (320.2) | 6.5 (4.4) |
| *Onset of MF (years)* | *≤1* | 85 | 63.7 (9.4) | 5.9 (2.8) | 6.0 (1.7) | 0.6 (0.3) | 2.4 (0.5) | 53 (62.4) | 616.6 (301.5) | 4.2 (2.9) |
|  | *>1* | 162 | 65.1 (8.6) | 8.6 (4.7) | 6.4 (2.1) | 3.8 (3.0) | 2.4 (0.5) | 98 (60.5) | 739.3 (323.3) | 7.4 (4.8) |
|  | *≤2* | 143 | 64.4 (9.1) | 6.0 (2.8) | 6.3 (2.0) | 1.0 (0.6) | 2.4 (0.5) | 92 (64.3) | 637.8 (316.0) | 4.5 (3.0) |
|  | *>2* | 104 | 64.9 (8.6) | 10.0 (4.9) | 6.3 (2.1) | 5.1 (3.1) | 2.4 (0.5) | 59 (56.7) | 778.6 (310.4) | 8.8 (5.0) |
| **Therapy-related subgroups** | | | | | | | | | | |
| *L-DOPA intakes (n)* | *<4* | 60 | 65.5 (9.4) | 5.6 (2.6) | 6.0 (1.7) | 1.7 (1.6) | 2.3 (0.6) | 40 (66.7) | 488.5 (198.4) | 4.3 (2.7) |
|  | *≥4* | 205 | 64.2 (8.7) | 8.2 (4.5) | 6.3 (2.1) | 3.1 (3.1) | 2.5 (0.5) | 120 (58.5) | 759.8 (325.8) | 6.9 (4.6) |
|  | *<5* | 132 | 65.4 (9.1) | 6.0 (3.0) | 6.0 (1.9) | 2.0 (1.9) | 2.4 (0.6) | 84 (63.6) | 560.4 (250.8) | 4.6 (3.0) |
|  | *≥5* | 133 | 63.6 (8.5) | 9.3 (4.7) | 6.5 (2.2) | 3.6 (3.5) | 2.4 (0.5) | 76 (57.1) | 835.3 (327.4) | 8.0 (4.9) |
|  | *<6* | 205 | 64.6 (9.1) | 6.9 (3.8) | 6.1 (2.0) | 2.4 (2.7) | 2.4 (0.5) | 127 (62.0) | 634.4 (285.3) | 5.6 (3.9) |
|  | *≥6* | 60 | 64.2 (8.0) | 10.1 (4.9) | 6.9 (2.2) | 3.9 (3.3) | 2.5 (0.5) | 33 (55.0) | 917.1 (346.4) | 8.8 (5.1) |
| *L-DOPA duration (years)* | *<4* | 97 | 63.5 (9.7) | 4.7 (1.8) | 6.2 (1.9) | 1.2 (0.9) | 2.4 (0.5) | 62 (63.9) | 592.4 (300.2) | 2.6 (0.8) |
|  | *≥4* | 168 | 65.1 (8.2) | 9.3 (4.4) | 6.3 (2.1) | 3.7 (3.2) | 2.4 (0.5) | 98 (58.3) | 759.6 (319.3) | 8.4 (4.2) |
|  | *<5* | 125 | 63.7 (9.5) | 4.9 (1.8) | 6.2 (1.9) | 1.3 (1.0) | 2.4 (0.5) | 79(63.2) | 587.5 (310.0) | 3.0 (1.1) |
|  | *≥5* | 140 | 65.3 (8.1) | 10.1 (4.3) | 6.3 (2.2) | 4.0 (3.6) | 2.4 (0.5) | 81 (57.9) | 797.4 (300.7) | 9.2 (4.1) |
|  | *<6* | 151 | 64.0 (9.4) | 5.1 (1.9) | 6.2 (1.8) | 1.5 (1.1) | 2.4 (0.5) | 95 (62.9) | 612.5 (319.0) | 3.4 (1.4) |
|  | *≥6* | 114 | 65.2 (8.1) | 11.0 (4.3) | 6.2 (2.3) | 4.4 (3.6) | 2.4 (0.5) | 65 (57.0) | 812.2 (290.5) | 10.1 (4.1) |
|  | *<7* | 174 | 64.1 (9.2) | 5.4 (2.0) | 6.2 (2.0) | 1.6 (1.2) | 2.4 (0.5) | 108 (62.1) | 631.5 (316.8) | 3.8 (1.6) |
|  | *≥7* | 91 | 65.3 (8.0) | 11.9 (4.2) | 6.2 (2.1) | 4.9 (3.8) | 2.5 (0.5) | 52 (57.1) | 826.3 (293.8) | 11.0 (4.1) |
|  | *<8* | 190 | 64.2 (9.1) | 5.7 (2.2) | 6.3 (2.1) | 1.7 (1.4) | 2.4 (0.5) | 117 (61.6) | 649.8 (314.0) | 4.1 (1.8) |
|  | *≥8* | 75 | 65.3 (8.0) | 12.5 (4.3) | 6.0 (2.0) | 5.2 (3.9) | 2.4 (0.6) | 43 (57.3) | 821.4 (311.6) | 11.8 (4.1) |
| *L-DOPA daily amount (mg)* | *<500* | 66 | 64.4 (10.1) | 6.0 (3.5) | 5.9 (2.0) | 2.1 (2.5) | 2.4 (0.6) | 39 (59.1) | 345.8 (78.8) | 4.6 (3.7) |
|  | *≥500* | 199 | 64.6 (8.4) | 8.2 (4.4) | 6.4 (2.1) | 3.0 (3.0) | 2.4 (0.5) | 121 (60.8) | 815.3 (284.8) | 6.9 (4.4) |
|  | *<600* | 103 | 64.7 (9.3) | 5.9 (3.0) | 5.8 (1.9) | 1.9 (2.2) | 2.4 (0.6) | 61 (59.2) | 404.0 (101.0) | 4.5 (3.2) |
|  | *≥600* | 162 | 64.4 (8.5) | 8.8 (4.6) | 6.5 (2.1) | 3.3 (3.1) | 2.4 (0.5) | 99 (61.1) | 885.6 (270.2) | 7.4 (4.6) |
|  | *<700* | 144 | 64.6 (9.1) | 6.5 (3.4) | 6.0 (2.0) | 2.1 (2.3) | 2.4 (0.6) | 80 (55.6) | 462.5 (126.6) | 5.1 (3.4) |
|  | *≥700* | 121 | 64.4 (8.5) | 9.0 (4.8) | 6.5 (2.1) | 3.5 (3.3) | 2.4 (0.5) | 80 (66.1) | 979.1 (250.9) | 7.8 (4.9) |
|  | *<800* | 176 | 64.4 (9.2) | 6.8 (3.7) | 6.1 (1.9) | 2.3 (2.6) | 2.4 (0.5) | 100 (56.8) | 512.6 (156.8) | 5.4 (3.7) |
|  | *≥800* | 89 | 64.7 (8.1) | 9.4 (4.8) | 6.6 (2.2) | 3.6 (3.3) | 2.4 (0.5) | 60 (67.4) | 1065.7 (238.7) | 8.1 (5.0) |
| *Use of L-DOPA only* | *Yes* | 68 | 65.6 (9.2) | 7.0 (4.3) | 6.6 (2.3) | 2.4 (2.6) | 2.5 (0.5) | 42 (61.8) | 730.3 (347.0) | 6.1 (4.4) |
|  | *No* | 197 | 64.2 (8.7) | 7.9 (4.3) | 6.1 (1.9) | 2.9 (3.0) | 2.4 (0.5) | 118 (59.9) | 687.4 (313.3) | 6.4 (4.4) |
| *Use of L-DOPA plus DA* | *Yes* | 180 | 64.5 (8.5) | 8.0 (4.3) | 6.1 (2.0) | 2.9 (3.0) | 2.4 (0.5) | 105 (58.3) | 689.2 (314.1) | 6.6 (4.4) |
|  | *No* | 85 | 64.6 (9.6) | 6.9 (4.1) | 6.5 (2.2) | 2.3 (2.5) | 2.4 (0.5) | 55 (64.7) | 717.8 (339.6) | 5.8 (4.2) |
| *Use of L-DOPA plus MAO-BI* | *Yes* | 57 | 62.8 (9.9) | 7.0 (4.0) | 5.9 (1.8) | 2.8 (3.0) | 2.3 (0.6) | 40 (70.2) | 604.2 (290.9) | 5.5 (4.2) |
|  | *No* | 208 | 65.0 (8.5) | 7.8 (4.3) | 6.3 (2.1) | 2.7 (2.8) | 2.5 (0.5) | 120 (57.7) | 724.2 (326.1) | 6.5 (4.4) |

Rows shaded in grey indicate variables generally associated with earlier disease course (shorter PD duration, lower H&Y staging, and shorter onset of MF; lower number of L-DOPA intakes, shorter duration of L-DOPA use, lower daily L-DOPA dose amount, and less use of adjunctive therapies), in comparison with matched unshaded rows. DA, dopamine agonist; H&Y, Hoehn and Yahr; L-DOPA, levodopa; MAO-BI, monoamine oxidase-B inhibitor; MF, motor fluctuations; OPC, opicapone; PD, Parkinson’s disease; SD, standard deviation.

**Supplementary Table 2. Baseline characteristics of PLC patient subgroups (Safety Set)**

| **Subgroup** | | **N** | **Baseline** | | | | | | | |
| --- | --- | --- | --- | --- | --- | --- | --- | --- | --- | --- |
|  |  |  | **Age (mean [SD] years)** | **Duration of PD (mean [SD] years)** | **Absolute OFF-time (mean [SD] h)** | **Time since onset of motor fluctuations (mean [SD] years)** | **H&Y staging at ON (mean [SD])** | **Male gender (n [%])** | **L-DOPA dose (mean [SD] mg)** | **Duration of L-DOPA therapy (mean [SD] years)** |
| **Disease-related subgroups** | | | | | | | | | | |
| *Duration of PD (years)* | *<6* | 103 | 63.3 (9.6) | 4.4 (0.9) | 6.1 (1.7) | 1.4 (1.1) | 2.3 (0.6) | 54 (52.4) | 542.4 (210.1) | 3.5 (1.4) |
|  | *≥6* | 154 | 62.6 (8.8) | 9.9 (3.6) | 6.2 (2.3) | 3.4 (2.3) | 2.4 (0.5) | 88 (57.1) | 796.2 (341.4) | 8.2 (3.5) |
|  | *<7* | 135 | 62.9 (9.2) | 4.9 (1.2) | 6.2 (1.8) | 1.6 (1.2) | 2.3 (0.6) | 70 (51.9) | 585.6 (267.1) | 3.9 (1.6) |
|  | *≥7* | 122 | 62.7 (9.1) | 10.9 (3.4) | 6.0 (2.3) | 3.7 (2.4) | 2.4 (0.5) | 72 (59.0) | 814.9 (332.6) | 9.0 (3.4) |
|  | *<8* | 156 | 62.9(9.2) | 5.2 (1.4) | 6.3 (1.9) | 1.7 (1.3) | 2.4 (0.6) | 84 (53.8) | 623.1 (316.3) | 4.2 (1.9) |
|  | *≥8* | 101 | 62.8 (9.1) | 11.6 (3.4) | 5.9 (2.4) | 3.9 (2.5) | 2.4 (0.5) | 58 (57.4) | 804.7 (296.3) | 9.5 (3.5) |
|  | *<9* | 181 | 62.7 (9.3) | 5.6 (1.7) | 6.4 (2.0) | 1.9 (1.4) | 2.4 (0.6) | 103 (56.9) | 658.8 (325.0) | 4.7 (2.1) |
|  | *≥9* | 76 | 63.1 (8.7) | 12.7 (3.2) | 5.6 (2.2) | 4.2(2.7) | 2.4 (0.5) | 39 (51.3) | 779.5 (294.9) | 10.2 (3.7) |
| *H&Y staging* | *<2.5* | 114 | 59.6 (9.7) | 7.1 (3.5) | 5.9 (1.8) | 2.4 (1.8) | 1.9 (0.3) | 62 (54.4) | 658.0 (329.7) | 6.0 (3.5) |
|  | *≥2.5* | 143 | 65.4 (7.7) | 8.2 (4.2) | 6.3 (2.3) | 2.8 (2.4) | 2.8 (0.3) | 80 (55.9) | 723.5 (311.3) | 6.6 (3.8) |
| *Onset of MF (years)* | *≤1* | 71 | 63.9 (9.4) | 5.8 (2.6) | 5.8 (1.8) | 0.6 (0.3) | 2.4 (0.5) | 37 (52.1) | 585.4 (274.6) | 4.4 (2.8) |
|  | *>1* | 174 | 62.3 (9.1) | 8.5 (4.1) | 6.3 (2.2) | 3.4 (2.1) | 2.4 (0.6) | 96 (55.2) | 742.3 (324.6) | 7.2 (3.6) |
|  | *≤2* | 125 | 64.0 (9.1) | 6.0 (2.6) | 6.2 (2.0) | 1.0 (0.6) | 2.3 (0.5) | 69 (55.2) | 637.9 (300.3) | 4.8 (2.7) |
|  | *>2* | 120 | 61.5 (9.1) | 9.5 (4.3) | 6.2 (2.2) | 4.3 (1.9) | 2.4 (0.6) | 64 (53.3) | 758.2 (326.4) | 8.0 (3.8) |
| **Therapy-related subgroups** | | | | | | | | | | |
| *L-DOPA intakes (n)* | *<4* | 51 | 65.5 (9.0) | 5.8 (2.7) | 6.1 (2.0) | 1.7 (1.7) | 2.3 (0.6) | 26 (51.0) | 468.9 (200.2) | 4.5 (2.9) |
|  | *≥4* | 206 | 62.2 (9.1) | 8.2 (4.0) | 6.2 (2.1) | 2.8 (2.2) | 2.4 (0.6) | 116 (56.3) | 750.3 (320.6) | 6.8 (3.7) |
|  | *<5* | 132 | 64.2 (10.0) | 6.6 (3.1) | 6.0 (2.1) | 2.0 (1.6) | 2.3 (0.6) | 71 (53.8) | 550.8 (227.9) | 5.4 (3.2) |
|  | *≥5* | 125 | 61.4 (8.1) | 8.9 (4.4) | 6.3 (2.0) | 3.3 (2.4) | 2.5 (0.6) | 71 (56.8) | 846.2 (334.8) | 7.3 (3.9) |
|  | *<6* | 197 | 63.3 (9.4) | 7.0 (3.6) | 6.1 (2.0) | 2.2(1.8) | 2.4 (0.6) | 105 (53.3) | 618.7 (256.2) | 5.6 (3.3) |
|  | *≥6* | 60 | 61.4 (8.1) | 9.9 (4.0) | 6.4 (2.2) | 4.0 (2.6) | 2.4 (0.6) | 37 (61.7) | 943.4 (381.8) | 8.5 (4.0) |
| *L-DOPA duration (years)* | *<4* | 78 | 62.6 (10.2) | 4.9 (2.8) | 5.8 (1.5) | 1.3 (1.0) | 2.3 (0.6) | 43 (55.1) | 517.6 (232.8) | 2.6 (0.9) |
|  | *≥4* | 179 | 62.9 (8.7) | 9.0 (3.7) | 6.3 (2.3) | 3.1 (2.3) | 2.4 (0.5) | 99 (55.3) | 771.5 (323.6) | 7.9 (3.2) |
|  | *<5* | 105 | 62.6 (9.7) | 5.2 (2.9) | 6.0 (1.7) | 1.4 (0.9) | 2.3 (0.6) | 58 (55.2) | 543.9 (237.7) | 3.1 (1.1) |
|  | *≥5* | 152 | 63.0 (8.8) | 9.4 (3.6) | 6.2 (2.3) | 3.4 (2.3) | 2.4 (0.5) | 84 (55.3) | 798.5 (329.7) | 8.5 (3.1) |
|  | *<6* | 142 | 62.8 (9.1) | 5.5 (2.6) | 6.2 (1.9) | 1.7 (1.2) | 2.4 (0.6) | 78 (54.9) | 582.2 (245.4) | 3.7 (1.4) |
|  | *≥6* | 115 | 62.8 (9.2) | 10.4 (3.5) | 6.1 (2.3) | 3.8 (2.5) | 2.4 (0.5) | 64 (55.7) | 833.1 (348.2) | 9.6 (2.9) |
|  | *<7* | 162 | 62.7 (9.0) | 5.7 (2.5) | 6.3 (2.0) | 1.8 (1.3) | 2.4 (0.6) | 86 (53.1) | 612.7 (290.0) | 4.0 (1.6) |
|  | *≥7* | 95 | 63.1 (9.4) | 11.2 (3.4) | 6.0 (2.3) | 3.9 (2.6) | 2.4 (0.5) | 56 (58.9) | 834.0 (323.6) | 10.2 (2.8) |
|  | *<8* | 181 | 62.6 (9.1) | 6.0 (2.7) | 6.2 (1.9) | 1.9 (1.4) | 2.4 (0.6) | 97 (53.6) | 642.9 (316.8) | 4.4 (1.8) |
|  | *≥8* | 76 | 63.4 (9.2) | 11.9 (3.2) | 5.9 (2.4) | 4.2 (2.7) | 2.4 (0.5) | 45 (59.2) | 817.3 (297.1) | 10.9 (2.6) |
| *L-DOPA daily amount (mg)* | *<500* | 69 | 64.0 (9.5) | 6.0 (2.7) | 6.0 (2.0) | 1.7 (1.5) | 2.2 (0.5) | 32 (46.4) | 353.1 (71.3) | 4.6 (3.1) |
|  | *≥500* | 188 | 62.4 (9.0) | 8.3 (4.1) | 6.2 (2.1) | 2.9 (2.3) | 2.4 (0.6) | 110 (58.5) | 819.8 (283.0) | 6.9 (3.7) |
|  | *<600* | 98 | 63.2 (9.4) | 6.1 (3.0) | 5.9 (2.0) | 2.0 (1.8) | 2.3 (0.6) | 45 (45.9) | 398.3 (92.7) | 4.7 (3.2) |
|  | *≥600* | 159 | 62.6 (9.0) | 8.7 (4.1) | 6.3 (2.1) | 3.0 (2.2) | 2.5 (0.5) | 97 (61.0) | 877.0 (270.8) | 7.3 (3.6) |
|  | *<700* | 139 | 63.3 (9.4) | 6.7 (3.5) | 6.1 (2.0) | 2.1 (1.9) | 2.3 (0.6) | 60 (43.2) | 462.6 (127.2) | 5.2 (3.2) |
|  | *≥700* | 118 | 62.2 (8.8) | 8.9 (4.1) | 6.2 (2.2) | 3.1 (2.3) | 2.4 (0.6) | 82 (69.5) | 967.6 (258.2) | 7.6 (3.8) |
|  | *<800* | 172 | 63.2 (9.4) | 7.0 (3.9) | 6.0 (2.0) | 2.3 (2.0) | 2.3 (0.6) | 80 (46.5) | 514.0 (156.1) | 5.4 (3.4) |
|  | *≥800* | 85 | 62.2 (8.7) | 9.1 (3.6) | 6.4 (2.3) | 3.3 (2.3) | 2.5 (0.6) | 62 (72.9) | 1059.7 (248.8) | 8.1 (3.6) |
| *Use of L-DOPA only* | *Yes* | 59 | 64.4 (9.8) | 6.2 (2.8) | 6.6 (2.3) | 1.8 (1.4) | 2.3 (0.6) | 36 (61.0) | 718.3 (359.1) | 5.1 (3.0) |
|  | *No* | 198 | 62.4 (8.9) | 8.2 (4.1) | 6.0 (2.0) | 2.9 (2.3) | 2.4 (0.6) | 106 (53.5) | 687.4 (308.8) | 6.7 (3.8) |
| *Use of L-DOPA plus DA* | *Yes* | 187 | 62.2 (8.8) | 8.1 (4.1) | 6.1 (2.0) | 2.9 (2.3) | 2.4 (0.6) | 98 (52.4) | 678.3 (304.7) | 6.6 (3.8) |
|  | *No* | 70 | 64.6 (9.7) | 6.5 (3.1) | 6.4 (2.2) | 1.9 (1.6) | 2.3 (0.6) | 44 (62.9) | 737.7 (358.5) | 5.4 (3.1) |
| *Use of L-DOPA plus MAO-BI* | *Yes* | 49 | 62.8 (9.0) | 8.3 (4.3) | 5.8 (2.0) | 2.4 (1.9) | 2.2 (0.6) | 32 (65.3) | 656.4 (325.2) | 6.0 (3.5) |
|  | *No* | 208 | 62.9 (9.2) | 7.6 (3.8) | 6.2 (2.1) | 2.6 (2.2) | 2.4 (0.5) | 110 (52.9) | 703.4 (319.6) | 6.4 (3.7) |

Rows shaded in grey indicate variables generally associated with earlier disease course (shorter PD duration, lower H&Y staging, and shorter onset of MF; lower number of L-DOPA intakes, shorter duration of L-DOPA use, lower daily L-DOPA dose amount, and less use of adjunctive therapies), in comparison with matched unshaded rows. DA, dopamine agonist; H&Y, Hoehn and Yahr; L-DOPA, levodopa; MAO-BI, monoamine oxidase-B inhibitor; MF, motor fluctuations; PD, Parkinson’s disease; PLC, placebo; SD, standard deviation.

**Supplementary Table 3. Change from baseline in total ON-time by subgroup (FAS)**

| **Subgroup** | | **OPC** | | **PLC** | | **OPC vs PLC**  **∆ (SE) change from baseline (min)** | **p-value** |
| --- | --- | --- | --- | --- | --- | --- | --- |
|  |  | **N** | **LS mean (SE)**  **change from baseline (min)** | **N** | **LS mean (SE)**  **change from baseline (min)** |  |  |
| **Disease-related subgroups** | | | | | | | |
| *Duration of PD (years)* | *<6* | 117 | 114.9 (14.3) | 102 | 28.7 (15.1) | **86.2 (20.5)** | <0.0001 |
|  | *≥6* | 145 | 114.7 (13.1) | 153 | 67.4 (12.6) | 47.4 (17.7) | 0.0076 |
|  | *<7* | 144 | 119.8 (12.9) | 133 | 36.3 (13.4) | **83.5 (18.2)** | <0.0001 |
|  | *≥7* | 118 | 108.5 (14.6) | 122 | 68.7 (14.1) | 39.8 (19.7) | 0.0444 |
|  | *<8* | 159 | 119.9 (12.3) | 154 | 42.9 (12.5) | **77.0 (17.1)** | <0.0001 |
|  | *≥8* | 103 | 106.6 (15.7) | 101 | 65.3 (15.5) | 41.4 (21.4) | 0.0541 |
|  | *<9* | 179 | 114.2 (11.7) | 179 | 46.6 (11.7) | **67.6 (16.0)** | <0.0001 |
|  | *≥9* | 83 | 115.8 (17.1) | 76 | 63.6 (17.7) | 52.2 (24.2) | 0.0313 |
| *H&Y staging* | *<2.5* | 113 | 131.2 (14.6) | 113 | 37.1 (14.4) | **94.1 (20.3)** | <0.0001 |
|  | *≥2.5* | 149 | 102.3 (12.9) | 142 | 63.4 (13.3) | 39.0 (17.8) | 0.0286 |
| *Onset of MF (years)* | *≤1* | 85 | 133.2 (17.2) | 71 | 55.1 (18.6) | **78.1 (25.0)** | 0.0019 |
|  | *>1* | 161 | 107.6 (12.5) | 172 | 53.1 (12.3) | 54.5 (17.0) | 0.0014 |
|  | *≤2* | 142 | 127.3 (13.2) | 125 | 46.9 (14.1) | **80.3 (18.9)** | <0.0001 |
|  | *>2* | 104 | 101.6 (15.6) | 118 | 61.1 (14.7) | 40.5 (20.9) | 0.0527 |
| **Therapy-related subgroups** | | | | | | | |
| *L-DOPA intakes (n)* | *<4* | 60 | 113.9 (20.0) | 51 | 48.3 (21.4) | **65.6 (28.9)** | 0.0236 |
|  | *≥4* | 202 | 115.0 (11.1) | 204 | 52.6 (11.1) | 62.4 (15.1) | <0.0001 |
|  | *<5* | 130 | 108.7 (13.6) | 130 | 42.3 (13.6) | **66.5 (18.8)** | 0.0004 |
|  | *≥5* | 132 | 121.0 (13.6) | 125 | 61.7 (14.1) | 59.3 (19.1) | 0.0020 |
|  | *<6* | 202 | 115.3 (11.0) | 195 | 48.4 (11.2) | **66.8 (15.3)** | <0.0001 |
|  | *≥6* | 60 | 113.1 (20.3) | 60 | 62.9 (20.6) | 50.2 (28.3) | 0.0767 |
| *L-DOPA duration (years)* | *<4* | 96 | 107.3 (15.9) | 77 | 25.3 (17.4) | **82.0 (23.3)** | 0.0005 |
|  | *≥4* | 166 | 119.4 (12.3) | 178 | 63.3 (11.8) | 56.0 (16.6) | 0.0008 |
|  | *<5* | 124 | 107.3 (13.8) | 104 | 27.0 (15.1) | **80.3 (20.1)** | <0.0001 |
|  | *≥5* | 138 | 121.8 (13.5) | 151 | 69.1 (12.7) | 52.6 (18.0) | 0.0036 |
|  | *<6* | 149 | 116.1 (12.7) | 140 | 34.8 (13.1) | **81.3 (17.8)** | <0.0001 |
|  | *≥6* | 113 | 112.5 (14.8) | 115 | 72.4 (14.6) | 40.1 (20.3) | 0.0487 |
|  | *<7* | 171 | 126.1 (11.8) | 160 | 36.9 (12.3) | **89.2 (16.6)** | <0.0001 |
|  | *≥7* | 91 | 92.2 (16.5) | 95 | 76.6 (15.9) | 15.6 (22.4) | 0.4855 |
|  | *<8* | 187 | 122.3 (11.4) | 179 | 40.7(11.7) | **81.7 (15.8)** | <0.0001 |
|  | *≥8* | 75 | 94.9 (18.2) | 76 | 77.7 (17.8) | 17.2 (24.9) | 0.4902 |
| *L-DOPA daily amount (mg)* | *<500* | 65 | 114.3 (19.1) | 68 | 32.8 (18.7) | **81.5 (26.3)** | 0.0020 |
|  | *≥500* | 197 | 114.9 (11.2) | 187 | 58.6 (11.5) | 56.3 (15.5) | 0.0003 |
|  | *<600* | 102 | 109.7 (15.2) | 97 | 25.6 (15.7) | **84.1 (21.5)** | 0.0001 |
|  | *≥600* | 160 | 118.3 (12.5) | 158 | 67.9 (12.4) | 50.4 (17.1) | 0.0033 |
|  | *<700* | 143 | 119.7 (13.0) | 138 | 40.0 (13.2) | **79.7 (18.1)** | <0.0001 |
|  | *≥700* | 119 | 108.9 (14.3) | 117 | 65.9 (14.4) | 42.9 (19.8) | 0.0306 |
|  | *<800* | 175 | 111.7 (11.8) | 170 | 43.6 (11.9) | **68.1 (16.4)** | <0.0001 |
|  | *≥800* | 87 | 121.2 (16.6) | 85 | 68.5 (16.8) | 52.7 (23.2) | 0.0236 |
| *Use of L-DOPA only* | *Yes* | 67 | 96.6 (18.9) | 59 | 22.8 (20.0) | **73.8 (27.1)** | 0.0066 |
|  | *No* | 195 | 121.1 (11.4) | 196 | 61.3 (11.3) | 59.9 (15.4) | 0.0001 |
| *Use of L-DOPA plus DA* | *Yes* | 178 | 115.8 (11.9) | 185 | 58.6 (11.6) | 57.2 (16.0) | 0.0004 |
|  | *No* | 84 | 112.3 (16.9) | 70 | 33.9 (18.4) | **78.4 (24.7)** | 0.0016 |
| *Use of L-DOPA plus MAO-BI* | *Yes* | 56 | 110.2 (20.6) | 49 | 32.5 (22.3) | **77.7 (29.7)** | 0.0090 |
|  | *No* | 206 | 115.9 (11.1) | 206 | 56.1 (10.9) | 59.8 (15.0) | <0.0001 |

Rows shaded in grey indicate variables generally associated with earlier disease course (shorter PD duration, lower H&Y staging, and shorter onset of MF; lower number of L-DOPA intakes, shorter duration of L-DOPA use, lower daily L-DOPA dose amount, and less use of adjunctive therapies), in comparison with matched unshaded rows. Values shown in bold indicate variables for which the difference in change from baseline in total ON-time for OPC 50 mg versus PLC (∆) was greater than that of the matched comparative row.

DA, dopamine agonist; H&Y, Hoehn and Yahr; L-DOPA, levodopa; LS, least square; MAO-BI, monoamine oxidase B inhibitor; MF, motor fluctuations; OPC, opicapone; PD, Parkinson’s disease; PLC, placebo; SE, standard error.

**Supplementary Table 4. Change from baseline in absolute ON-time with troublesome dyskinesia by subgroup (FAS)**

| **Subgroup** | | **OPC** | | **PLC** | | **OPC vs PLC**  **∆ (SE) change from baseline (min)** | **p-value** |
| --- | --- | --- | --- | --- | --- | --- | --- |
|  |  | **n** | **LS mean (SE)**  **change from baseline (min)** | **n** | **LS mean (SE)**  **change from baseline (min)** |  |  |
| **Disease-related subgroups** | | | | | | | |
| *Duration of PD (years)* | *<6* | 117 | 7.9 (7.9) | 102 | 3.9 (8.3) | **4.0 (11.3)** | 0.7222 |
|  | *≥6* | 145 | 21.3 (7.2) | 153 | 4.2 (6.9) | 17.1 (9.7) | 0.0791 |
|  | *<7* | 144 | 10.8 (7.1) | 133 | 1.1 (7.4) | **9.7 (10.0)** | 0.3301 |
|  | *≥7* | 118 | 21.0 (8.0) | 122 | 7.4 (7.7) | 13.6 (10.8) | 0.2101 |
|  | *<8* | 159 | 7.5 (6.8) | 154 | -0.4 (6.9) | **7.9 (9.4)** | 0.3997 |
|  | *≥8* | 103 | 28.1 (8.6) | 101 | 11.1 (8.5) | 17.0 (11.7) | 0.1465 |
|  | *<9* | 179 | 9.1 (6.4) | 179 | -2.0 (6.4) | 11.1 (8.8) | 0.2060 |
|  | *≥9* | 83 | 28.5 (9.4) | 76 | 17.9 (9.7) | **10.7 (13.3)** | 0.4214 |
| *H&Y staging* | *<2.5* | 113 | 14.8 (8.0) | 113 | 3.8 (7.9) | **11.1 (11.1)** | 0.3201 |
|  | *≥2.5* | 149 | 15.3 (7.1) | 142 | 4.0 (7.3) | 11.3 (9.8) | 0.2463 |
| *Onset of MF (years)* | *≤1* | 85 | 15.3 (9.5) | 71 | 6.7 (10.2) | **8.6 (13.8)** | 0.5316 |
|  | *>1* | 161 | 16.4 (6.9) | 172 | 4.3 (6.8) | 12.1 (9.4) | 0.2000 |
|  | *≤2* | 142 | 11.3 (7.3) | 125 | 7.0 (7.8) | **4.3 (10.4)** | 0.6835 |
|  | *>2* | 104 | 22.5 (8.6) | 118 | 2.7 (8.1) | 19.8 (11.5) | 0.0858 |
| **Therapy-related subgroups** | | | | | | | |
| *L-DOPA intakes (n)* | *<4* | 60 | 2.1 (11.0) | 51 | 0.9 (11.7) | **1.1 (15.8)** | 0.9432 |
|  | *≥4* | 202 | 19.1 (6.1) | 204 | 4.7 (6.0) | 14.4 (8.3) | 0.0822 |
|  | *<5* | 130 | 2.5 (7.5) | 130 | 6.9 (7.4) | **-4.4 (10.3)** | 0.6695 |
|  | *≥5* | 132 | 27.9 (7.5) | 125 | 0.7 (7.6) | 27.1 (10.4) | 0.0095 |
|  | *<6* | 202 | 15.4 (6.0) | 195 | 5.2 (6.2) | **10.2 (8.4)** | 0.2239 |
|  | *≥6* | 60 | 14.6 (11.1) | 60 | 0.0 (11.3) | 14.6 (15.5) | 0.3471 |
| *L-DOPA duration (years)* | *<4* | 96 | 8.9 (8.7) | 77 | 15.3 (9.5) | **-6.4 (12.7)** | 0.6163 |
|  | *≥4* | 166 | 18.6 (6.8) | 178 | -1.2 (6.5) | 19.8 (9.1) | 0.0295 |
|  | *<5* | 124 | 9.0 (7.6) | 104 | 7.6 (8.3) | **1.4 (11.0)** | 0.8970 |
|  | *≥5* | 138 | 20.9 (7.4) | 151 | 1.6 (7.0) | 19.3 (9.9) | 0.0511 |
|  | *<6* | 149 | 5.6 (7.0) | 140 | 6.4 (7.2) | **-0.8 (9.7)** | 0.9345 |
|  | *≥6* | 113 | 28.2 (8.1) | 115 | 1.1 (8.0) | 27.1 (11.1) | 0.0148 |
|  | *<7* | 171 | 11.5 (6.5) | 160 | 3.5 (6.8) | **8.0 (9.1)** | 0.3776 |
|  | *≥7* | 91 | 22.4 (9.1) | 95 | 5.0 (8.7) | 17.5 (12.3) | 0.1553 |
|  | *<8* | 187 | 13.9 (6.3) | 179 | 1.5 (6.4) | 12.4 (8.7) | 0.1527 |
|  | *≥8* | 75 | 19.0 (10.0) | 76 | 10.2 (9.8) | **8.8 (13.6)** | 0.5193 |
| *L-DOPA daily amount (mg)* | *<500* | 65 | 2.2 (10.5) | 68 | 0.1 (10.3) | **2.2 (14.4)** | 0.8797 |
|  | *≥500* | 197 | 19.6 (6.2) | 187 | 5.5 (6.3) | 14.1 (8.5) | 0.0977 |
|  | *<600* | 102 | 5.2 (8.3) | 97 | -4.6 (8.6) | **9.8 (11.8)** | 0.4093 |
|  | *≥600* | 160 | 22.0 (6.9) | 158 | 9.4 (6.8) | 12.6 (9.4) | 0.1792 |
|  | *<700* | 143 | 8.5 (7.1) | 138 | -4.6 (7.2) | 13.0 (9.9) | 0.1888 |
|  | *≥700* | 119 | 23.5 (7.8) | 117 | 14.0 (7.9) | **9.5 (10.8)** | 0.3805 |
|  | *<800* | 175 | 10.4 (6.5) | 170 | 0.5 (6.5) | **9.9 (9.0)** | 0.2696 |
|  | *≥800* | 87 | 25.2 (9.1) | 85 | 11.2 (9.2) | 13.9 (12.7) | 0.2737 |
| *Use of L-DOPA only* | *Yes* | 67 | 7.3 (10.4) | 59 | -2.6 (10.9) | **9.9 (14.9)** | 0.5081 |
|  | *No* | 195 | 18.1 (6.2) | 196 | 6.2 (6.2) | 11.8 (8.5) | 0.1622 |
| *Use of L-DOPA plus DA* | *Yes* | 178 | 16.1 (6.5) | 185 | 5.0 (6.3) | **11.1 (8.8)** | 0.2083 |
|  | *No* | 84 | 13.5 (9.2) | 70 | 1.5 (10.1) | 12.0 (13.5) | 0.3762 |
| *Use of L-DOPA plus MAO-BI* | *Yes* | 56 | 20.6 (11.3) | 49 | 6.3 (12.2) | 14.3 (16.3) | 0.3823 |
|  | *No* | 206 | 13.8 (6.1) | 206 | 3.5 (6.0) | **10.3 (8.2)** | 0.2131 |

Rows shaded in grey indicate variables generally associated with earlier disease course (shorter PD duration, lower H&Y staging, and shorter onset of MF; lower number of L-DOPA intakes, shorter duration of L-DOPA use, lower daily L-DOPA dose amount, and less use of adjunctive therapies), in comparison with matched unshaded rows. Values shown in bold indicate variables for which the difference in change from baseline in absolute ON-time with troublesome dyskinesia for OPC 50 mg versus PLC (∆) was less than that of the matched comparative row. DA, dopamine agonist; H&Y, Hoehn and Yahr; L-DOPA, levodopa; LS, least square; MAO-BI, monoamine oxidase B inhibitor; MF, motor fluctuations; OPC, opicapone; PD, Parkinson’s disease; PLC, placebo; SE, standard error.
